# Supplementary figures and images for: Deficiency in Galectin-3, -8, and -9 impairs immunity to chronic Mycobacterium tuberculosis infection but not acute infection with multiple intracellular pathogens
Source: PLoS Pathog. 2023 Jun 23;19(6):e1011088. doi: 10.1371/journal.ppat.1011088 (PMC10325092; doi:10.1371/journal.ppat.1011088)

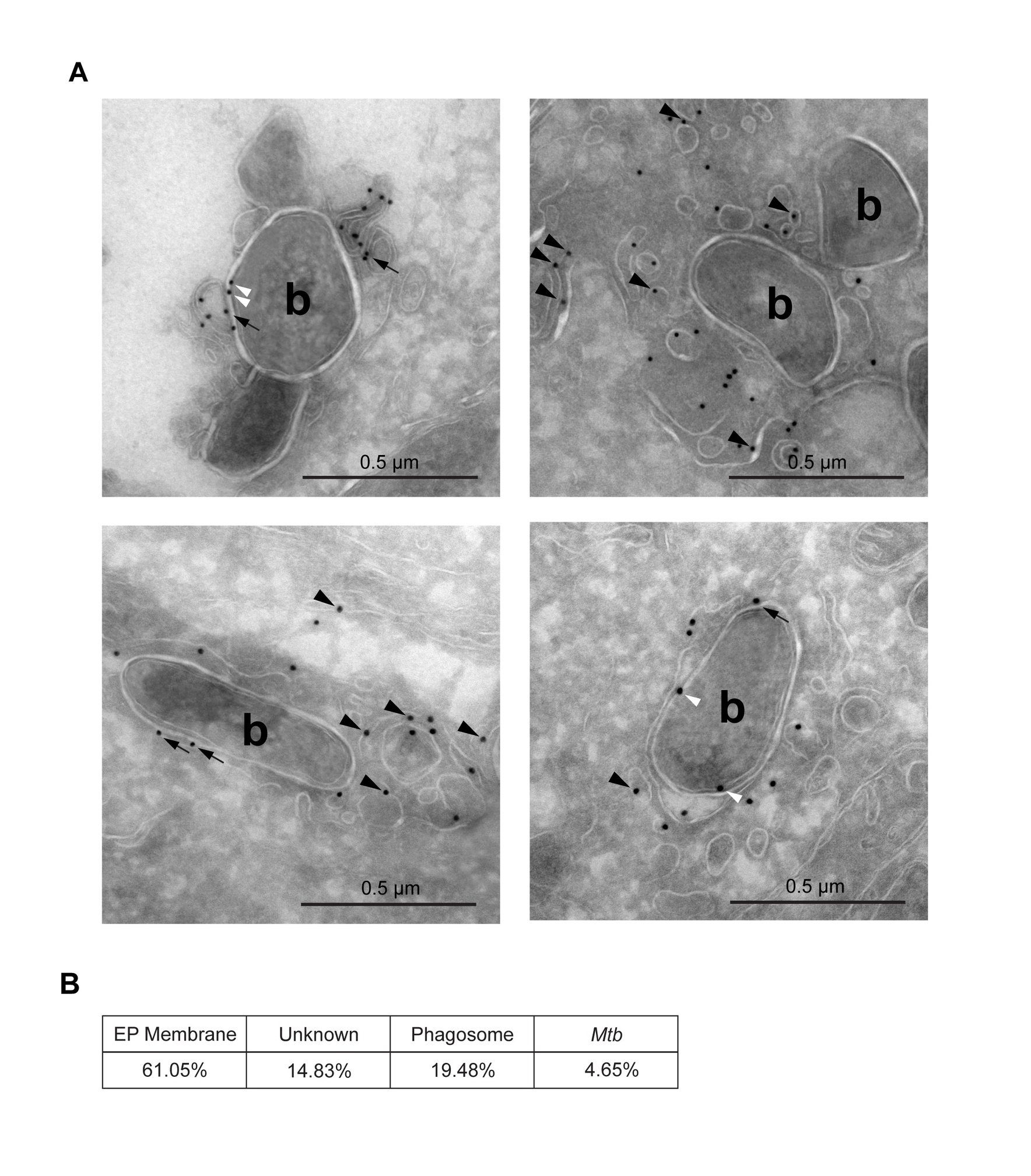

Supplement: S1 Fig — (A) Cryosections of RAW 264.7 cells stably expressing Gal-9-FLAG infected with WT Mtb (MOI = 1) 6 hours post-infection. Gal-9 localization to extra-phagosomal membranes (black arrowheads), phagosomes (black arrows), and Mtb (white arrowheads); b, bacteria. Four representative micrographs are shown from a dataset of 26 total images. Scale bar = 0.5 μm. (B) Quantification of (A) for Gal-9-FLAG localization to indicated structures; EP membrane, extra-phagosomal membranes. Values are a percent of total Gal-9-FLAG puncta in the dataset. (TIFF) [file ppat.1011088.s001.tiff]

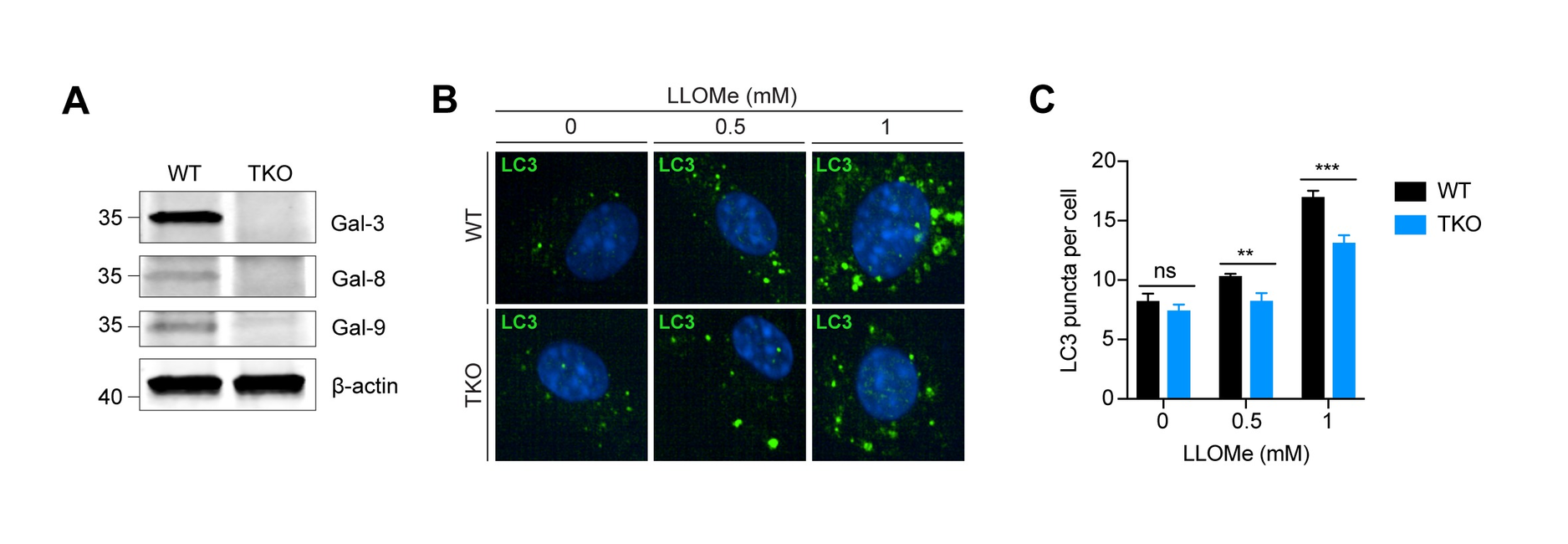

Supplement: S2 Fig — (A) Immunoblots of bone marrow-derived macrophage (BMM) lysates from WT and TKO mice (strain 2) probed for indicated proteins. (B) Confocal microscopy of WT or TKO BMMs treated with LLOMe at the indicated concentrations for 2 hours and immunostained for LC3. (C) Quantification of (B) for LC3 puncta per cell. Figures are representative of three independent experiments (B, C). An average of 400 cells were analyzed per technical replicate (C). Error bars represent SD from four technical replicates, and **p<0.01, ***p<0.001 by unpaired t-test. (TIFF) [file ppat.1011088.s002.tiff]

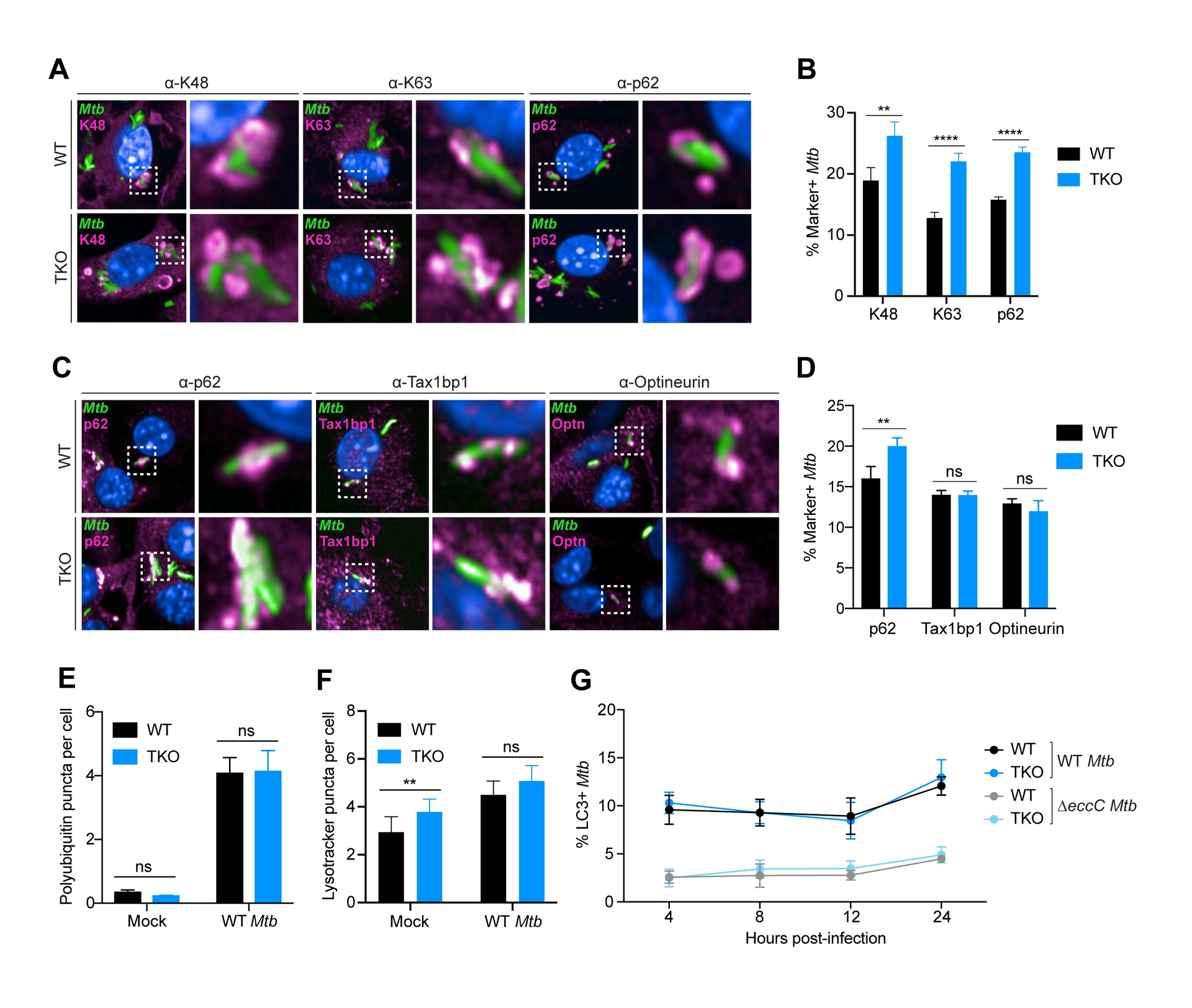

Supplement: S3 Fig — (A) Confocal microscopy of WT or TKO BMMs infected with Mtb-GFP (MOI = 2) 8 hours post-infection and immunostained for K48- or K63-linked polyubiquitin, or p62. (B) Quantification of (A) for Mtb-GFP colocalization with indicated markers. (C) Confocal microscopy of WT or TKO BMMs infected with Mtb-GFP (MOI = 2) 8 hours post-infection and immunostained for p62, Tax1bp1, or Optineurin. (D) Quantification of (C) for Mtb-GFP colocalization with indicated markers. (E) Quantification of polyubiquitin puncta 8 hours post-infection in mock- or Mtb-GFP-infected (MOI = 2) WT or TKO BMMs. (F) Quantification of Lysotracker puncta 8 hours post-infection in mock- or Mtb-GFP-infected (MOI = 2) WT or TKO BMMs. (G) Quantification of LC3 colocalization with WT or ΔeccC Mtb (MOI = 2) at indicated time points. Figures are representative of two (A-D) independent experiments or represent one (G) or two (E, F) independent experiments. An average of 747 (A, B), 926 (C, D), 1464 (E, F), and 429 (G) cells were analyzed per technical replicate. Error bars represent SD from three (E, F) or four (A-D, G) technical replicates, and **p<0.01, ****p<0.0001 by unpaired t-test. (TIFF) [file ppat.1011088.s003.tiff]

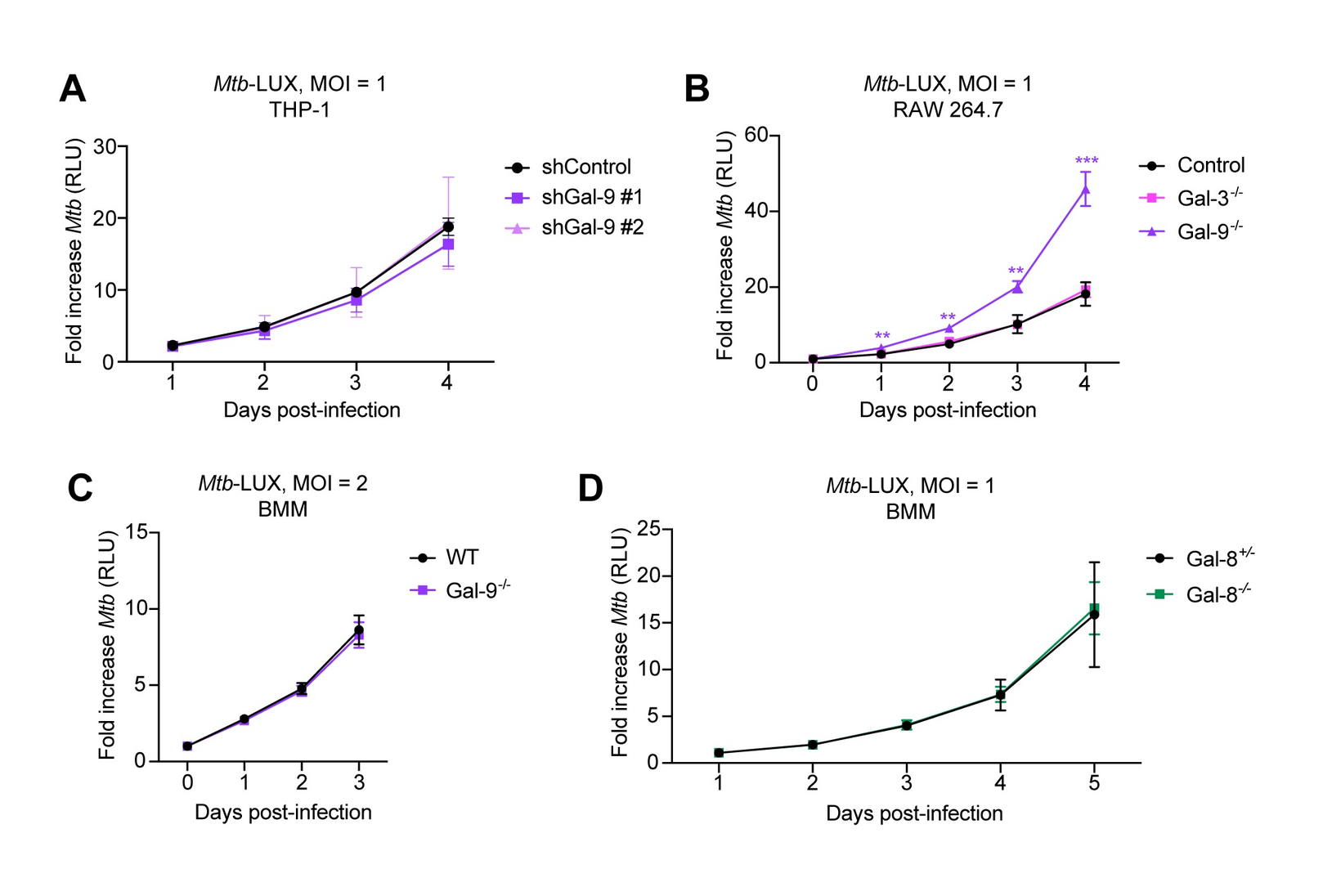

Supplement: S4 Fig — RLU-fold change of (A) control or Gal-9 knockdown shRNA THP-1 cells infected with Mtb-LUX (MOI = 1), (B) control, Gal-3-/-, or Gal-9-/- RAW 264.7 cells infected with Mtb-LUX (MOI = 1), (C) WT or Gal-9-/- BMMs infected with Mtb-LUX (MOI = 2), or (D) Gal-8+/- or Gal-8-/- BMMs infected with Mtb-LUX (MOI = 1). Figures are representative of two (B, D) or three (C) independent experiments, or represent four independent experiments (A). Error bars represent SD from three technical replicates, and **p<0.01, ***p<0.001 by unpaired t-test. (TIFF) [file ppat.1011088.s004.tiff]

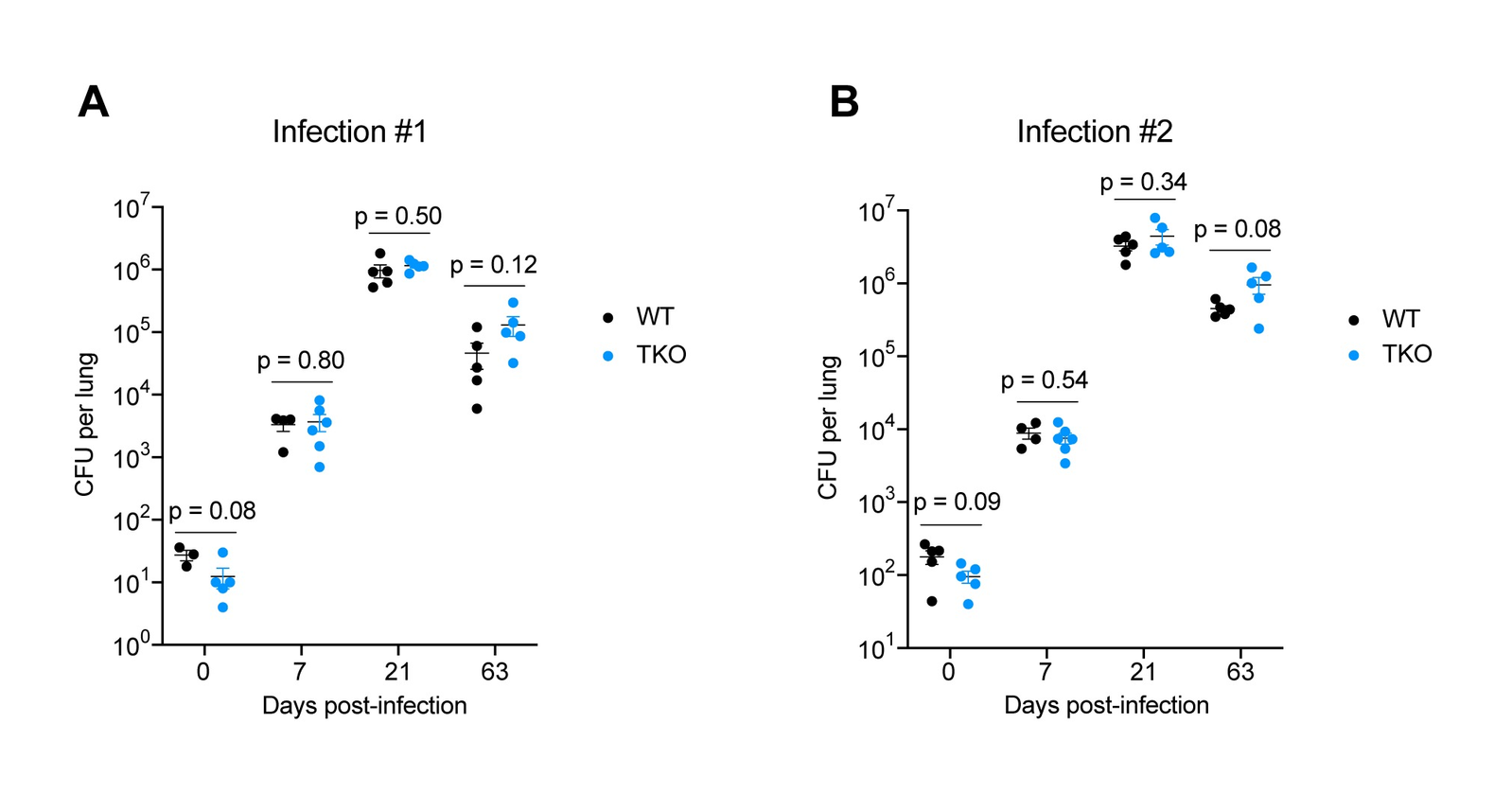

Supplement: S5 Fig — WT and TKO female mice were aerosol infected with (A) ~18 CFUs or (B) ~136 CFUs of Mtb Erdman and bacterial loads in lungs were enumerated by plating for CFU at indicated time points. n = 3–5 mice per genotype. Figures represent one experiment. Bars represent the mean, error bars represent SEM, and p-values were determined by unpaired t-test. (TIFF) [file ppat.1011088.s005.tiff]

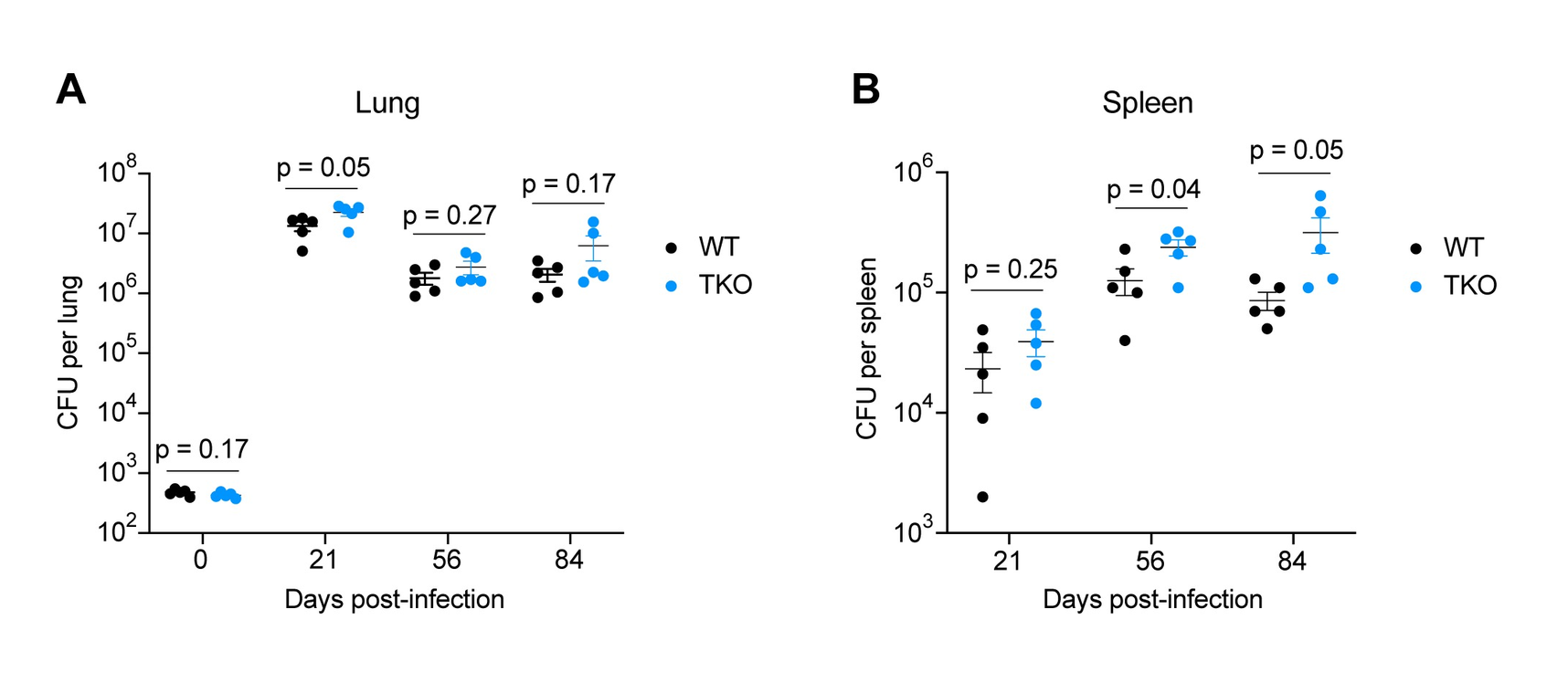

Supplement: S6 Fig — (A) WT and TKO male mice were aerosol infected with ~450 CFUs of Mtb Erdman and bacterial loads in lungs were enumerated by plating for CFU at indicated time points. n = 5 mice per genotype. (B) Same as in (A) but bacterial loads in spleens. Figures represent one experiment. Bars represent the mean, error bars represent SEM, and p-values were determined by unpaired t-test. (TIFF) [file ppat.1011088.s006.tiff]

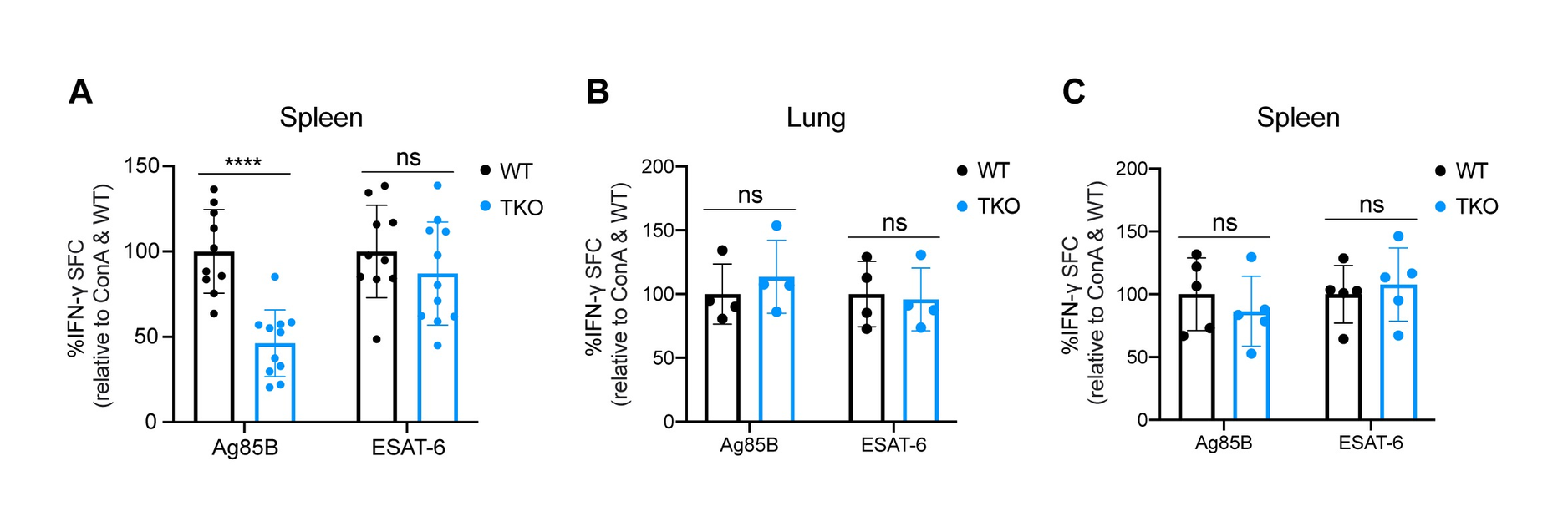

Supplement: S7 Fig — (A) WT and TKO mice were aerosol infected with ~100 CFUs of Mtb Erdman and splenocytes were isolated for IFN-γ ELISpot to enumerate Mtb-specific T cells. Mtb-specific spot-forming cells (SFC) responding to re-stimulation in vitro with Mtb peptides are shown for each condition relative to Concanavalin A (ConA) total T cells and WT. n = 10–11 mice per genotype. (B) Same as in (A) but ELISpot performed with lung single cell suspension. n = 4 mice per genotype. (C) ELISpot on splenocytes following high-dose aerosol inoculation (~800 CFUs). n = 5 mice per genotype. Figures represent data from three (A) independent experiments or one (B, C) independent experiment. Error bars represent SD, and ****p<0.0001 by unpaired t-test. (TIFF) [file ppat.1011088.s007.tiff]
